# Desquamation after Scarlet Fever

**Published:** 1895-04-20

**Authors:** 


### Desquamation after Scarlet Fever.

In the scientific investigation of disease one must be prepared not only to accept new facts but to throw away old traditions, and although we are far from saying that the matter is ripe for decision one way or the other it is clear that the question how far the "scales" thrown off in the desquamation period of scarlet fever are infectious is one which requires to be carefully investigated afresh, and in regard to which we must all be prepared to accept a verdict according to the evidence, however adverse it may be to the cruder teaching of earlier times.

No one, probably, will dispute that the scales of epidermis which have been part of the patient during the course of such an infectious disease as scarlet fever will, like his clothes, harbour infection, and, like his clothes, will continue to harbour it until they are washed or in some manner disinfected, but that a patient who has lost all signs of disease and is completely well in every respect, except a certain roughness of skin and superabundant shedding of epidermis should still continue to be looked upon as infectious so long as this roughness persists is another matter, and one the importance of which is strongly forcing itself on the attention of both hospital managers and the public.

The sanitary authorities in many districts, and the Asylums Board in London have with a more or less light heart taken up the task of treating all and sundry who are suffering from infectious fevers. That in itself is a by no means inconsiderable undertaking. But as this is done, not as a charity to the individual affected, but with the definite object of preventing the spread of the disease, it is obviously necessary to keep up the isolation so long as the patient remains infectious. Now, as estimated by the period of desquamation, this is a long time, and under the constantly-increasing sense of responsibility resulting from the application of the sanitary law is becoming longer. Notwithstanding all the care which is taken, fresh developments of scarlet fever now and again arise on the return from hospital of patients who have been discharged as free from infection, and the belief in the infectiousness of the desquamation has now led to such prolonged detention that the whole scheme of hospital isolation is becoming seriously imperilled. The hospital authorities find their acute cases swamped by an enormous crowd of convalescents "peeling," but otherwise well; while people outside who would be glad enough to have their children nursed and treated while they are ill, hesitate to send them away to play, maybe for months, among the heterogenous crew which a public isolation hospital receives.

No one thing could more facilitate the working of the isolation hospital system than a shortening of the weary period of waiting for the end of desquamation after scarlet fever. Whatever then may be the explanation it is impossible not to admit the importance of a fact which was lately communicated to the Epidemiological Society by Dr. Joseph Priestley, who stated that when on the outbreak of small-pox in Leicester 120 cases of scarlet fever were sent home in every stage of desquamation to make way for small-pox cases, in no single instance did a second case occur at any of the houses to which these desquamating patients returned, although such an event was carefully watched for for three months.

Considerable interest then attaches to Dr. Priestley's report of the cases treated last year at Leicester, according to the plans advocated a few years ago by Dr. Curgenven, viz., by inunction with eucalyptus oil and spraying the throat and nasal cavities with the same material. One hundred and twenty cases were so treated, and were compared with 161 cases as nearly as possible equal to them in severity, with the result that there seemed to be in favour of the eucalyptus a lower death rate, a shorter stay in hospital, slightly fewer complications of a serious nature, and fewer "return" cases; and it is worth noting that although twenty-three cases were discharged while the feet were still peeling, the five "return" cases were all quite smooth when they left the hospital.

While, however, Dr. Priestley's observations give some reason for hoping that the prolonged detention in hospital entailed by inordinately slow desquamation may in future be lessened, it is important that people should not rush to the extreme of believing that by any local application to the skin the infectiousness of the fever in its acute stage can be so nullified as to make it desirable that scarlet fever should be treated at home. Everything points to the probability that scarlet fever is in essence an infectious throat disease, and that the destruction of epidermis is a secondary affair. Undoubtedly this epidermis, although dead, is charged with infection, but whether after the acute stage it has any power of renewing its infectiveness, if it can once be destroyed, is another matter, and as to this it ought not to be difficult to obtain some definite information. But during the active progress of the fever it is from the throat and nose and their discharges that infection is mostly to be feared, and during that period it is not to be expected that either inunction or the use of disinfecting spray will prevent its spread.

The satisfactory isolation of scarlet fever would, however, be enormously facilitated if the period of detention could be shortened, and it is much to be wished that some such method of dealing with the skin as is suggested by Dr. Priestley, supplemented by some means of disinfecting the upper air passages may prove efficacious.

